# Gaps in Serologic Immunity against Contemporary Swine-Origin Influenza A Viruses among Healthy Individuals in the United States

**DOI:** 10.3390/v13010127

**Published:** 2021-01-18

**Authors:** Joshua N. Lorbach, Theresa Fitzgerald, Carolyn Nolan, Jacqueline M. Nolting, John J. Treanor, David J. Topham, Andrew S. Bowman

**Affiliations:** 1 Department of Veterinary Preventive Medicine, College of Veterinary Medicine, The Ohio State University, Columbus, OH 43210, USA; lorbach.5@osu.edu (J.N.L.); nolting.4@osu.edu (J.M.N.); 2 Department of Microbiology and Immunology, University of Rochester Medical Center, Rochester, NY 14627, USA; Theresa_Fitzgerald@URMC.Rochester.edu (T.F.); Carolyn_Nolan@URMC.Rochester.edu (C.N.); david_topham@urmc.rochester.edu (D.J.T.); 3 Department of Medicine, University of Rochester Medical Center, Rochester, NY 14627, USA; John_Treanor@urmc.rochester.edu

**Keywords:** influenza A virus, orthomyxovirus, serology, immunology, swine, zoonoses

## Abstract

Influenza A Viruses (IAV) in domestic swine (IAV-S) are associated with sporadic zoonotic transmission at the human–animal interface. Previous pandemic IAVs originated from animals, which emphasizes the importance of characterizing human immunity against the increasingly diverse IAV-S. We analyzed serum samples from healthy human donors (*n* = 153) using hemagglutination-inhibition (HAI) assay to assess existing serologic protection against a panel of contemporary IAV-S isolated from swine in the United States (*n* = 11). Age-specific seroprotection rates (SPR), which are the proportion of individuals with HAI ≥ 1:40, corresponded with lower or moderate pandemic risk classifications for the multiple IAV-S examined (one H1-δ1, one H1-δ2, three H3-IVA, one H3-IVB, one H3-IVF). Individuals born between 2004 and 2013 had SPRs of 0% for the five classified H3 subtype IAV-S, indicating youth may be particularly predisposed to infection with these viruses. Expansion of existing immunologic gaps over time could increase likelihood of future IAV-S spillover to humans and facilitate subsequent sustained human-to-human transmission resulting in disease outbreaks with pandemic potential.

## 1. Introduction

Influenza A Virus (IAV) is a pathogen of global concern based on its history and future potential to cause millions of human deaths if pandemic spread occurs [1]. Four true IAV pandemics have occurred since 1918, each of which was started by zoonotic transmission of the responsible virus from animal reservoirs to humans [1,2,3]. The most recent influenza pandemic occurred in 2009 when a novel H1N1 subtype IAV arose in swine and eventually breached the species barrier to infect humans [2,4]. Pre-existing immunity conferred through natural infection or vaccination with earlier H1N1 subtype human seasonal IAVs was insufficient to prevent worldwide spread of this newly-emerged 2009 virus [5]. The ensuing pandemic was responsible for an estimated 284,000 deaths and a marked shift in disease burden wherein the vast majority of deaths occurred in individuals younger than age 65 [5]. Identifying IAVs with the potential for pandemic behavior is essential to protecting public health.

Clinical influenza caused by spillover of swine-linage IAV (IAV-S) from pigs to humans is called “variant” influenza [6]. Human seasonal IAVs, in contrast, are viruses endemic to humans worldwide that become established following emergence of a pandemic IAV. Introduction of these human seasonal IAVs to swine via reverse zoonosis occurs regularly [7,8]. These viruses can be maintained in swine where they experience relatively little immunologic pressure. As human population immunity changes over time, this may permit spillback of viruses previously introduced to swine from humans. Spillback events have been recognized years after a human IAV was introduced to swine. In 2016, multiple human cases of variant influenza were caused by an H3 (human-like) IAV-S transmitted in association with exhibition swine contact. This virus was introduced by humans to commercial swine in 2010–2011 and identified through surveillance activities shortly thereafter. It eventually spread to exhibition swine in the five years between introduction and spillback [9,10,11].

Swine are infected with diverse IAVs, frequently leading to emergence of novel reassortant viruses capable of infecting humans [6,12]. Current IAV-S diversity in the US has largely been driven by the introduction of the triple reassortant internal gene (TRIG) constellation in the 1990s, reverse zoonotic transmission of IAVs from humans to swine, and continuous antigenic drift of surface glycoproteins [13,14]. The result has been the co-circulation of at least ten distinct hemagglutinin (HA) genetic clades in US swine, including: H3-IV-[A through–F], α-H1, β-H1, γ-H1, δ1-H1, δ2-H1, and H1pdm (Supplementary Figure S1) [12,15,16]. In general, IAV diversity in US swine is maintained in commercial swine herds with bottlenecking at the exhibition swine–human interface where the majority of zoonotic transmission occurs [17]. Movement of viruses between geographically distant swine populations is supported by an extensive, poorly understood transmission network [17].

The zoonotic potential of IAV and the connection of swine with the most recent influenza pandemic makes this species an important target for IAV surveillance [18,19]. The numerous variant influenza cases recognized annually in the United States provide mounting evidence that spillover of IAV from swine to humans was not limited to the 2009 pandemic, and multiple IAVs circulating in swine (IAV-S) can cause zoonotic infection of individuals at the human–animal interface [20,21,22]. Since 2010, 465 cases of variant influenza caused by IAV-S have been documented in the US (CDC FluView Interactive Novel Influenza A Virus Infections Tool), including a large outbreak of variant influenza associated with swine contact in 2012-2013 wherein over 90% of cases were children [10]. There has not been sustained human-to-human transmission following these zoonotic IAV transmission events. However, continued spillover of IAV-S to humans raises concern about the potential for subsequent sustained transmission in the human population following future zoonotic infection events [22,23].

A novel IAV-S that causes clinical disease in humans may be assigned a quantified risk of pandemic behavior using multifactorial predictive models such as the Influenza Risk Assessment Tool (IRAT; Centers for Disease Control and Prevention) or the Tool for Influenza Pandemic Risk Assessment (TIPRA; World Health Organization) [24,25]. These tools characterize the overall threat of viruses using several risk elements that are based on information about the virus, human population, animal hosts. Pre-established criteria are used to evaluate each risk element and scores corresponding to perceived risk are assigned by experts. TIPRA for example, categorizes the risk for each element with a three-tier risk stratification system (lower, moderate, and higher risk). Both TIPRA and IRAT consider existing cross-protective immunity in the human population as a critical risk factor during these assessments [24,25]. The overall prevalence and functional importance of human cross-reactive anti-IAV antibodies (Ab) is not well-established, but existing humoral immunity (or lack thereof) is likely to mitigate or enhance the morbidity and mortality of influenza during the course of a pandemic [26].

Human serologic immunity against IAVs maintained in swine provides a way to partially estimate the risk of zoonotic transmission into the human population, as well as pandemic risk. Introduction of seasonal IAVs from humans into swine populations occurs frequently, maintaining past human-lineage IAVs in this animal reservoir while population anti-IAV immunity changes significantly [6,7]. The prevalence of cross-reactive Ab in humans providing protection from pandemic influenza remains poorly understood. Because human anti-IAV Ab is expected to vary by age and natural exposure history, it is important to quantify and characterize serum Ab in multiple age groups [26,27]. The vast majority of swine-to-human IAV transmission has occurred in children [22,28], suggesting that age-related immunologic gaps exist and may permit novel viruses to become established in humans following zoonosis. Identifying and understanding existing deficiencies in human immunity against infection with IAV-S is critical to pandemic preparedness efforts and the implementation of risk mitigation strategies aimed at preventing future zoonotic IAV transmission. To characterize human immunity against a panel of contemporary IAV-S, we performed a cross-sectional analysis of serum samples from healthy human donors using hemagglutination-inhibition (HAI) assay.

## 2. Materials and Methods

Thirteen IAVs were selected for assessment including 11 IAV-S strains [A/Swine/MN/2010(H3N2), A/Swine/OH/2011(H1N2), A/Swine/OH/2011(H3N2), A/Swine/MN/2012(H3N2), A/Swine/NE/2012(H3N2), A/Swine/NC/2013(H3N2), A/Swine/SD/2013(H1N1), A/Swine/IN/2013(H1N1), A/Swine/OH/2013(H1N2), A/Swine/OH/2016(H3N2), A/Swine/MI/2016(H3N2)] and two human seasonal IAV vaccine strains [A/CA/2009(H1N1), A/TX/2012(H3N2)]. Complete name, hemagglutinin (HA) subclade nomenclature, and GenBank accession number (HA segment) of study viruses are listed in Supplementary Table S1. Swine-origin IAVs were chosen to reflect the diversity of major H1 and H3 clades circulating in US commercial and exhibition swine after 2009 based on H1 and H3 classification systems [25,29]. H1 and H3 subtype human IAVs that have recently circulated as seasonal strains and were incorporated into vaccines were chosen to serve as reference in HAI assays. Low-passage IAV isolates were obtained from our IAV surveillance project and supplemented with viruses from the United States Department of Agriculture (USDA) repository. [30,31]. HA phylogenies including subclade classification of study viruses are shown for H1 subtype viruses in Supplementary Figure S2 and H3 subtype viruses are shown in Supplementary Figure S3. Additional data for subclade classification were obtained from the NIAID Influenza Research Database (IRD) [32]. Phylogenetic trees were estimated using RaxML with a general time reversible substitution model plus the gamma parameter of rate heterogeneity [33].

Human serum samples were previously collected with informed consent between 18 January 2010 and 14 March 2014 from healthy, non-immunosuppressed individuals (*n* = 153) enrolled in a sero-epidemiological cohort study at the University of Rochester Medical Center under RSRB protocol #58358. Individuals were between 2 and 78 years of age at the time of sample collection. No additional information (e.g., sex, race, vaccination status) was examined for the samples used in this study. Hemagglutination inhibition (HAI) tests were performed with turkey red blood cells. Individual serum samples were pretreated with receptor-destroying enzyme (Denka Seiken Co Ltd., Tokyo, Japan) and tested following serial two-fold dilution with a starting dilution of 1:10. HAI assays were performed using “V” bottom microtiter plates as previously described [34].

HAI titers < 1:10 were assigned a nominal value of 1:5 for descriptive and analytic statistics based on the assay limit of detection. Geometric mean titers (GMT) were calculated using natural-log-transformation. Individual seroprotection was defined as an HAI titer ≥ 1:40, corresponding with 50% reduction in individual disease risk [35]. Seroprotection rate (SPR) was defined as the proportion of individuals with an HAI titer ≥ 1:40. For some antigens, positive control ferret sera were available and used in the assay, including A/TX/2012 (H3N2) and A/Swine/MN/2010. Negative controls consisted of antigen-alone wells and the reagent control contained phosphate-buffered saline with red blood cells.

Descriptive and analytic statistics were performed with Stata v14 (Stata Corporation College Station, TX, USA). Age groups were constructed to compare immunity within distinct subsets of the cohort sample; two methods of binning were examined (Table 1). The first was constructed using age cutoffs reported in studies of anti-IAV immunity and international guidelines for assessing IAV pandemic potential [24,36]. The resulting age groups were defined as “juvenile” (< 18 years old, *n* = 44), “adult” (18 to 49 years old, *n* = 65), and “senior” (≥ 50 years old, *n* = 44). In the second method of binning, groups were constructed by decade of birth starting with the earliest birth year represented in the sample set (birth year 1934–1943, *n* = 10; 1944–1953, *n* = 15; 1954–1963, *n* = 22; 1964–1973, *n* = 14; 1974–1983, *n* = 14, 1984–1993, *n* = 34; 1994–2003, *n* = 25; 2004–2013, *n* = 19). Some individual serum specimens were unable to be used in HAI assays for all study viruses (Supplementary Table S2). Risk categorizations for study viruses were defined by TIPRA guidelines and assigned based on within-age group SPR: “lower risk”, SPR ≥ 30% in all age groups except juveniles; “moderate risk”, SPR ≥ 30% only in seniors; “higher risk”, SPR < 10% in all age groups [24]. The lower boundary of SPR 95% confidence intervals were also considered when they fell below the cutoffs of individual risk strata. Pearson’s chi-squared test was used to assess independence of SPRs (HAI titer ≥ 1:40) between age groups. A significance threshold of *p* < 0.05 was used.

## 3. Results

### 3.1. H1 Subtype IAV-S

**Figure 1:**
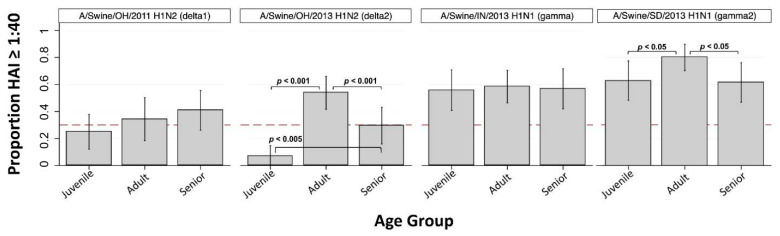
Seroprotection rate (SPR) against H1 subtype IAV-S by age group (*n* = 3). Bar height indicates proportion of individuals with HAI antibody titer > 1:40 (seroprotection rate; SPR) within Juvenile (<18 years old), Adult (18 to 49 years old), and Senior (≥50 years old) age groups. Dashed red line at *y* = 0.3 corresponds to SPR of 30%. Vertical spikes and caps indicate 95% confidence interval of the proportion. Significant difference in proportions between groups are indicated when present by horizontal brackets and p-values (Pearson’s chi-squared).

Virus-specific seroprotection rate (SPR) and geometric mean HAI titer (GMT) are shown for juveniles, adults, seniors, and overall in Table 2 and Table 3, respectively. H1-subtype IAV-S were associated with overall SPRs ranging from 32.2% to 65.4% and overall GMTs ranging from 17 to 58. Overall GMTs for A/swine/OH/2011 (H1-δ1) and A/swine/OH/2013 (H1-δ2) were 17 and 18, respectively, and overall SPRs were 32.2% and 33.3%. SPRs for A/swine/OH/2011 (H1-δ1) were below 41% for juveniles, adults, and seniors. The juvenile SPR < 30% indicates TIPRA risk classification as “lower risk” for this H1-δ1 IAV-S. However, the adult SPR was near the 30% cutoff and if the lower 95% confidence interval (CI) of the adult SPR is considered this virus is elevated to “moderate risk” classification (Table 2). The juvenile SPR was 7.0% for A/swine/OH/2013 (H1-δ2), significantly lower than the adult SPR (53.8%, *p* < 0.001) and senior SPR (29.5%, *p* < 0.005) (Figure 1). This H1-δ2 IAV-S was thus classified as “lower risk” based on the within-group SPRs < 30% (Table 2). While the senior SPR was <30% for this virus and significantly lower than the adult SPR (*p* < 0.001), this did not impact risk classification.

The overall GMT against A/swine/IN/2013 (H1-γ) was 35, and the overall SPR was 53.5%. SPRs among juveniles, adults, and seniors were not significantly different from each other and did not indicate risk classification (Figure 1). The GMT against A/swine/SD/2013 (H1-γ2) was 58, and the overall SPR was 65.4%. Juvenile SPR (62.8%) and senior SPR (61.4%) were both significantly lower than adult SPR (80.0%) (juvenile vs. adult, *p* < 0.05; senior vs. adult *p* < 0.05) (Figure 1). Similar to the H1-γ virus, within-group SPRs did not indicate risk classification for the H1-γ2 IAV-S.

### 3.2. H3 Subtype IAV-S

**Figure 2:**
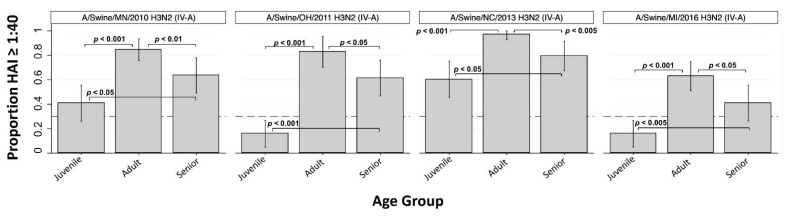
Seroprotection rate (SPR) against H3 cluster IV-A IAV-S by age group (*n* = 3). Bar height indicates proportion of individuals with HAI antibody titer >1:40, which is the seroprotection rate (SPR) within Juvenile (<18 years old), Adult (18 to 49 years old), and Senior (≥50 years old) age groups. Dashed red line at *y* = 0.3 corresponds to SPR of 30%. Vertical spikes and caps indicate 95% confidence interval of the proportion. Significant difference in proportions between groups are indicated (when present) by horizontal brackets and *p*-values (Pearson’s chi-squared).

Among the four H3 cluster IV-A IAVs, overall GMTs ranged from 33 to 120 and overall SPRs ranged from 42.5% to 77.7%. Within-age group SPRs for H3 cluster IV-A viruses consistently demonstrated highest seroprotection in adults followed by seniors and then juveniles (Figure 2). Juvenile SPR was significantly lower compared to both adults and seniors for all four cluster IV-A viruses. As shown in Figure 2, within-group SPRs for A/swine/OH/2011 and A/swine/MI/2016 along with the lower 95% CI bounds of the SPR for A/swine/MN/2010 indicated “lower risk” classification for these three viruses. Although A/swine/NC/2013 also was associated with significantly decreased seroprotection in juveniles as compared to adults (*p* < 0.001) and seniors (*p* < 0.05), the within-group SPRs were all > 60% and did not meet criteria for risk classification (Table 2). The differences in SPRs among the H3 cluster IV-A IAVs is likely due to expansion and evolution of cluster IV-A over time.

**Figure 3:**
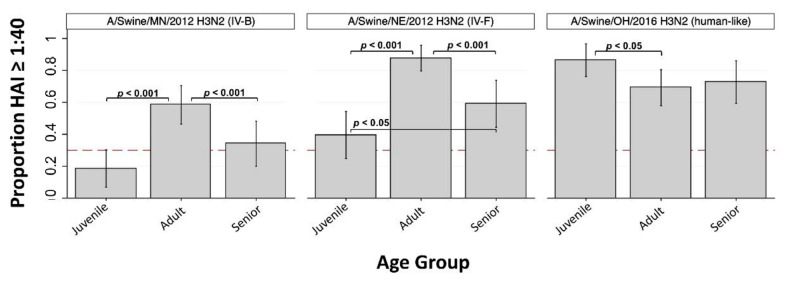
Seroprotection rate (SPR) against H3 cluster IV-B, IV-F, and human-like IAV-S by age group (*n* = 3). Bar height indicates proportion of individuals with HAI antibody titer ≥ 1:40 seroprotection rate (SPR) within Juvenile (<18 years old), Adult (18 to 49 years old), and Senior (≥ 50 years old) age groups. Dashed red line at *y* = 0.3 corresponds to SPR of 30%. Vertical spikes and caps indicate 95% confidence interval of the proportion. Significant difference in proportions between groups are indicated (when present) by horizontal brackets and p-values (Pearson’s chi-squared).

The GMT against A/swine/MN/2012 (H3 cluster IV-B) was 19, and the overall SPR was 39.0%. Seroprotection rates were significantly lower in seniors (34.1%, *p* < 0.001) and juveniles (18.6%, *p* < 0.001) compared to adults (58.5%), and this virus was classified as “lower risk” based on within-group SPRs (Figure 3, Table 2).

The GMT against A/swine/NE/2012 (H3 cluster IV-F) was 60, and the overall SPR was 63.3%. Seroprotection rates were significantly lower in seniors (59.1%, *p* < 0.001) and juveniles (39.5%, *p* < 0.001) compared to adults (87.7%), and the juvenile SPR was significantly lower than the senior SPR (*p* < 0.05) (Figure 3). This virus was also classified as “lower risk” based on within-group SPRs (Table 2). The GMT against A/swine/OH/2016 (H3 human-like) was 78, and the overall SPR was 69.1%. Seroprotection against this virus was significantly higher for juveniles (86.4%) compared to adults (69.2%, *p* < 0.05).

### 3.3. Seroprotection within Decades of Birth

Seroprotection rates were also examined by decades of birth (Supplementary Figures S4 and S5). There were five IAV-S for which the youngest decade of birth (individuals born 2004–2013, *n* = 19) SPR was 0%. This included three H3 cluster IV-A viruses (A/swine/MN/2010, A/swine/OH/2011, A/swine/MI/2016), one H3 cluster IV-B virus (A/swine/MN/2012), and one H3 cluster IV-F virus (A/swine/NE/2012). While significant differences in SPR between decades of birth may exist, small sample sizes were a limitation when comparing these groups.

### 3.4. Human Seasonal IAVs

**Figure 4:**
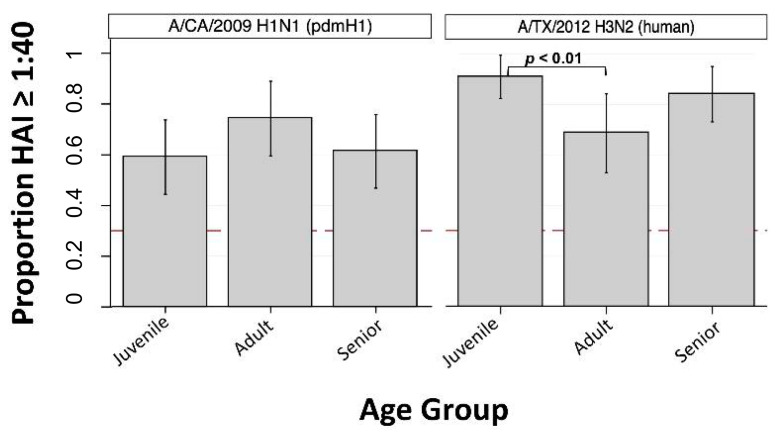
Seroprotection rate (SPR) against pdmH1 and H3 human seasonal IAVs by age group (*n* = 3). Bar height indicates proportion of individuals with HAI antibody titer >1:40, the seroprotection rate (SPR) within Juvenile (<18 years old), Adult (18 to 49 years old), and Senior (≥50 years old) age groups. Dashed red line at *y* = 0.3 corresponds to SPR of 30%. Vertical spikes and caps indicate 95% confidence interval of the proportion. Significant difference in proportions between groups are indicated (when present) by horizontal brackets and *p*-values (Pearson’s chi-squared).

The overall GMT against the human seasonal IAV A/CA/2009 (H1-pdm) was 49, and the overall SPR was 62.2%. No significant differences in SPR were observed between age groups for A/CA/2009 (Figure 4). The GMT against the human seasonal IAV A/TX/2012 (H3-human) was 104, and the SPR against this virus was 73.3%. Seroprotection against A/TX/2012 (H3-human) was significantly higher in juveniles (90.9%) compared to adults (68.6%, *p* < 0.01) (Figure 4).

## 4. Discussion

We have identified significant gaps in human cross-protective immunity against multiple contemporary IAV-S. Once a human IAV becomes established in pigs, the swine and human lineages of the strain will diverge over time. Given the relatively short lifespan of pigs, swine production creates a continuous supply of immunologically susceptible pigs, which allows the virus to persist in the swine population. Meanwhile, concurrent continued circulation of the virus in the human population will result in antigenic drift with corresponding changes to human immunity. Over time, older people retain immunity to the progenitor virus, but younger people, who were not exposed to the progenitor virus, only have immunity against an antigenically drifted version and are susceptible to the progenitor virus sequestered in the swine population. This was highlighted during 2009 H1N1 influenza pandemic when older age groups had better antibody response than younger people [38]. As more immunologically susceptible people are born each day, immunological gaps are in a continual state of expansion [39]. The magnitude of the gaps will influence the outcome of future zoonotic transmission events at the human–animal interface.

There have been numerous introductions of human seasonal H1 and H3 IAVs into swine, which have given rise to a diverse set of IAV lineages in swine (Supplementary Figure S1). Lineages such as α-H1 have been endemic in swine for upwards of a century, whereas newer introductions have been circulating in swine for only a few years. The H1-γ and H1-γ2 lineages are genetically and antigenically similar to the H1N1pdm09 strain that has become established in humans [15], which explains why we detected high SPRs to these swine strains (Figure 1). Likewise, we observed a high SPR for A/Swine/OH/2016, which represents one of the most recent IAV spillovers from humans to swine (Figure 3). The H1-δ1 IAV-S, A/swine/OH/2011 (H1N2), evaluated here represents a former human seasonal H1 subtype IAV introduced from people to pigs nearly two decades ago. Although this virus was replaced in humans after the emergence of H1N1pdm09, it was able to persist in swine. Currently, immunity is lacking against this H1-δ1 virus in all age groups in our cohort, with decreasing SPR from the oldest to youngest individuals. Only 33.3% (SPR, crude) of the serum specimens we examined had a protective HAI titer against this virus, and within-group SPRs indicated classification as “moderate risk” based on serology alone using TIPRA cutoffs [24]. As the proportion of the population born after this virus was circulating in human continues to increase, so does the threat to public health.

Gaps in immunity specific to certain age groups indicate youth are particularly predisposed to infection with multiple IAV-S currently circulating in swine in the US. The SPR for juveniles born 2004–2013 was 0% for five IAV-S examined in our study. All five of these viruses were H3 subtype IAV-S, with clusters IV-A, IV-B, and IV-F represented. The absence of serologic protection in this youngest age group corroborates previous epidemiologic reports of variant influenza associated with swine at agricultural exhibitions and highlights an increased risk of IAV transmission to youth at the human–animal interface. The majority of sequenced H3N2 subtype IAV isolates from 2009–2016 in US swine were classified as cluster IV-A [12]. Interestingly, seroprotection against the A/swine/NC/2013 cluster IV-A virus was elevated in all age groups and did not indicate risk classification, though the reason for this discrepancy was not apparent.

As the human population ages, the proportion of people lacking cross-protective immunity to these prevalent IAV-S will increase, which means the pandemic threat of viruses maintained in the swine population needs to be continually reevaluated. During an outbreak of variant influenza caused by an H3N2 (cluster IV) IAV-S in 2012–2013 there were 306 human cases identified, over 90% of which were children [10]. The apparent age disparity in regard to disease burden seen among cases of variant influenza may be explained by the large number of youth participants at fairs relative to older individuals, or by heightened concern for signs of influenza-like illness in children. However, differences in childhood IAV exposure history seem likely to explain relative differences in serologic immunity [6,12,40]. Insufficient serologic immunity in juveniles seen for several H3 subtype IAV-S in our study suggest the health burden amongst youth may be disproportionately elevated should the next pandemic IAV be of this subtype [34]. While our cohort represents a geographically restricted subset of the population, we expect that similar trends in national immunity would exist in populations of youth with limited exposure to swine.

Past influenza pandemics have been caused by IAVs with little to no pre-existing human immunity, a feature derived from major shifts in IAV antigenicity occurring in animal reservoirs. This was seen in 2009 when the novel reassortant influenza virus A(H1N1) pdm09 emerged from commercial swine in Mexico [2,3,7,26]. Complete risk assessment to quantify pandemic potential requires multiple datasets addressing validated risk elements including the ability of an IAV-S to infect humans. Since cross-protective anti-IAV immunity is a crucial barrier to IAV-S transmission from swine to humans, gaps in serologic immunity fundamentally elevates pandemic risk. Presently there are insufficient immunologic barriers to prevent spillover of several IAV-S to humans.

We did not examine mucosal immunity or cell-mediated immunity because of the type and quantity of human biospecimens available for this study. Antibodies directed against IAV antigens other than HA, such as the neuraminidase (NA) protein, can also provide protection from infection. Still, HAI titers are commonly used as a correlate of immunologic protection against infection with IAVs in risk assessment tools designed and implemented by the WHO and the CDC. The cohort of patients represented by the serum samples examined here is not reflective of the US population at large. To minimize the impact of this limitation, overall SPRs reported for each virus have been standardized using recent US population age distribution data. Occupational or natural exposure of cohort members to swine is not known, though such contact is assumed to be minimal. Individuals frequently having contact with swine may possess higher serologic immunity against the swine origin viruses examined here, making interspecies transmission less likely. Additional studies to understand anti-IAV immunity unique to individuals, especially youth, in frequent contact with swine are ongoing. Regardless, IAV spillover events at the swine-human interface continue to occur, and the immunologic gaps identified here could potentiate human-to-human spread of IAV once it has overcome the species barrier.

Continued surveillance is paramount to identifying IAV-S that threaten public health. Effectively recognizing cases of variant influenza and thoroughly assessing emerging IAVs for pandemic risk are not always possible. Adoption of proactive strategies, including improved biosecurity procedures and vaccination of swine, are necessary to prevent future zoonotic transmission of IAV-S with pandemic potential. Repeated cross-sectional serosurveillance in youth may act as an early warning system to identify IAV-S capable of pandemic spread. Expanding the assessment of existing human serologic cross-protection against current endemic IAV-S in additional US and global populations will further inform pandemic preparedness efforts and may identify new subpopulations at increased risk for infection.

## Figures and Tables

**Table 1:** Study serum sample age distribution shown by decade of birth (*n* = 8) and age group (*n* = 3).

		No. Samples
**Birth year**	1934–1943	10
	1944–1953	15
	1954–1963	22
	1964–1973	14
	1974–1983	14
	1984–1993	34
	1994–2003	25
	2004–2013	19
**Age group ^a^**	Juvenile ^b^	44
	Adult ^c^	65
	Senior ^d^	44
**Overall**		153

^a^ Age group cutoffs were constructed based on those utilized in the Tool for Influenza Pandemic Risk Assessment (TIPRA) [24]. ^b^ Individuals < 18 years old; ^c^ Individuals 18–49 years old; ^d^ Individuals ≥ 50 years old.

**Table 2:** Seroprotection rates ^a^ against study influenza A viruses by age group ^b^ and overall.

		HAI ≥ 1:40 [Proportion (95% CI)]			
Virus	HA Subclade	Juvenile ^c^	Adult ^d^	Senior ^e^	Overall ^f^
A/Swine/OH/2011 (H1N2)	delta1	25.0% ** (12.1, 37.9)	34.3% ** (18.3, 50.2)	40.9% (26.2, 55.6)	32.2%
A/Swine/OH/2013 (H1N2)	delta2	7.0% * (0, 14.7)	53.8% (41.6, 66.1)	29.5% (15.9, 43.2)	33.3%
A/Swine/IN/2013 (H1N1)	gamma	55.8% (40.8, 70.8)	58.5% (46.4, 70.5)	56.8% (42.0, 71.6)	53.5%
A/Swine/SD/2013 (H1N1)	gamma2	62.8% (48.2, 77.4)	80.0% (70.2, 89.8)	61.4% (46.8, 75.9)	65.4%
A/CA/2009 (H1N1)	pdmH1	59.1% (44.4, 73.8)	74.3% (59.6, 89.0)	61.4% (46.8, 75.9)	62.2%
A/Swine/MN/2010 (H3N2)	C_IV-A	40.9% * (26.2, 55.6)	84.6% (75.8, 93.5)	63.6% (49.3, 78.0)	63.7%
A/Swine/OH/2011 (H3N2)	C_IV-A	15.9% * (5.0, 26.8)	82.9% (70.2, 95.5)	61.4% (46.8, 75.9)	57.2%
A/Swine/NC/2013 (H3N2)	C_IV-A	60.5% (45.7, 75.3)	96.9% (92.7, 100)	79.5% (67.5, 91.6)	77.7%
A/Swine/MI/2016 (H3N2)	C_IV-A	15.9% * (5.0, 26.8)	63.1% (51.3, 74.9)	40.9% (26.2, 55.6)	42.5%
A/Swine/MN/2012 (H3N2)	C_IV-B	18.6% * (6.8, 30.4)	58.5% (46.4, 70.5)	34.1% (19.9, 48.3)	39.0%
A/Swine/NE/2012 (H3N2)	C_IV-F	39.5% * (24.7, 54.3)	87.7% (79.6, 95.7)	59.1% (44.4, 73.8)	63.3%
A/Swine/OH/2016 (H3N2)	human-like	86.4% (76.1, 96.6)	69.2% (57.9, 80.5)	72.7% (59.4, 86.0)	69.1%
A/TX/2012 (H3N2)	human	90.9% (82.3, 99.5)	68.6% (53.0, 84.2)	84.1% (73.2, 95.0)	73.3%

^a^ Seroprotection rate (SPR) indicated by % individuals with HAI ≥ 1:40. ^b^ Age groups constructed based on those utilized in the Tool for Influenza Pandemic Risk Assessment (TIPRA) [24]; ^c^ Individuals < 18 years old; ^d^ Individuals 18–49 years old; ^e^ Individuals ≥ 50 years old; ^f^ SPRs (%) reported for the overall group have been standardized using 2018 single-year United States population estimates reported by the U.S. Census Bureau [37]; * Within-age group SPR or lower bound of 95% confidence interval satisfies TIPRA risk classification criteria for “Lower risk”; ** Within-age group SPR or lower bound of 95% confidence interval satisfies TIPRA risk classification criteria for “Moderate risk”.

**Table 3:** Geometric mean titers (GMT) ^a^ against study influenza A viruses by age group ^b^ and overall.

		GMT [Mean (95% CI)]			
Virus	Subclade	Juvenile ^c^	Adult ^d^	Senior ^e^	Overall
A/Swine/OH/2011 (H1N2)	delta1	13 (9, 18)	17 (11, 27)	24 (17, 34)	18 (14, 22)
A/Swine/OH/2013 (H1N2)	delta2	8 (6, 10)	31 (20, 48)	14 (10, 21)	17 (13, 21)
A/Swine/IN/2013 (H1N1)	gamma	35 (20, 61)	39 (25, 60)	30 (20, 44)	35 (27, 46)
A/Swine/SD/2013 (H1N1)	gamma2	49 (29, 85)	91 (62, 134)	34 (23, 51)	58 (44, 75)
A/CA/2009 (H1N1)	pdmH1	47 (30, 74)	78 (45, 137)	36 (24, 54)	49 (38, 65)
A/Swine/MN/2010 (H3N2)	C_IV-A	19 (13, 29)	136 (95, 197)	29 (20, 41)	50 (38, 64)
A/Swine/OH/2011 (H3N2)	C_IV-A	11 (8, 15)	110 (64, 187)	39 (27, 56)	33 (25, 44)
A/Swine/NC/2013 (H3N2)	C_IV-A	46 (29, 72)	267 (200, 357)	94 (63, 139)	120 (94, 152)
A/Swine/MI/2016 (H3N2)	C_IV-A	9 (7, 12)	48 (31, 73)	25 (16, 37)	24 (19, 31)
A/Swine/MN/2012 (H3N2)	C_IV-B	10 (8, 14)	30 (22, 42)	18 (13, 24)	19 (16, 23)
A/Swine/NE/2012 (H3N2)	C_IV-F	19 (12, 31)	163 (109, 244)	42 (27, 66)	60 (45, 80)
A/Swine/OH/2016 (H3N2)	human-like	95 (62, 146)	77 (48, 121)	64 (43, 95)	78 (60, 100)
A/TX/2012 (H3N2)	human	179 (119, 269)	71 (40, 127)	83 (57, 120)	104 (80, 136)

^a^ GMTs are reported as the inverse ratio of the titer; individual titers below 10 (HAI < 1:10) were transformed to a nominal titer of 5 for mean titer calculations; ^b^ Age groups constructed based on those utilized in the Tool for Influenza Pandemic Risk Assessment (TIPRA) [24]; ^c^ Individuals < 18 years old; ^d^ Individuals 18–49 years old; ^e^ Individuals ≥ 50 years old.

## Data Availability

Not applicable.

## Supplementary Materials

The following are available online at https://www.mdpi.com/1999-4915/13/1/127/s1. Supplementary Figure S1. Contemporary United States Swine Influenza A Virus H1 and H3 Hemagglutinin Segments; Supplementary Figure S2: Phylogenetic relationships of hemagglutinin sequences for H1-subtype IAV study strains; Supplementary Figure S3: Phylogenetic relationships of hemagglutinin sequences for H3-subtype IAV study strains; Supplementary Figure S4: Group seroprotection rate (8 decades of birth) for H1 subtype IAVs; Supplementary Figure S5: Group seroprotection rate (8 decades of birth) for H3 subtype IAVs; Supplementary Table S1: Study virus nomenclature and GenBank accession numbers (hemagglutinin segment); Supplementary Table S2: Number of serum samples analyzed by HAI assay within individual age groups and overall.

## References

[1] Horimoto T., Kawaoka Y. (2005). Influenza: Lessons from past pandemics, warnings from current incidents. Nat. Rev. Microbiol..

[2] Mena I., I Nelson M., Quezada-Monroy F., Dutta J., Cortes-Fernández R., Lara-Puente J.H., Castro-Peralta F., Cunha L.F., Trovão N.S., Lozano-Dubernard B. (2016). Origins of the 2009 H1N1 influenza pandemic in swine in Mexico. eLife.

[3] Kilbourne E.D. (2006). Influenza Pandemics of the 20th Century. Emerg. Infect. Dis..

[4] Smith G.J.D., Vijaykrishna D., Bahl J., Lycett S.J., Worobey M., Pybus O.G., Ma S.K., Cheung C.L., Raghwani J., Bhatt S. (2009). Origins and evolutionary genomics of the 2009 swine-origin H1N1 influenza A epidemic. Nat. Cell Biol..

[5] Dawood F.S., Iuliano A.D., Reed C., Meltzer M.I., Shay D.K., Cheng P.Y., Widdowson M.A. (2012). Estimated global mortality associated with the first 12 months of 2009 pandemic in-fluenza A H1N1 virus circulation: A modelling study. Lancet Infect. Dis..

[6] Van Reeth K., Vincent A.L., Zimmerman J.J., Karriker L.A., Ramirez A., Schwartz K.J., Stevenson G.W., Zhang J. (2019). Influenza Viruses. Diseases of Swine.

[7] Nelson M.I., Vincent A.L. (2015). Reverse zoonosis of influenza to swine: New perspectives on the human–animal interface. Trends Microbiol..

[8] Nelson M.I., Stratton J., Killian M.L., Janas-Martindale A., Vincent A.L. (2015). Continual Reintroduction of Human Pandemic H1N1 Influenza A Viruses into Swine in the United States, 2009 to 2014. J. Virol..

[9] Schicker R.S., Rossow J.A., Eckel S., Fisher N., Bidol S., Tatham L., Matthews-Greer J., Sohner K., Bowman A.S., Avrill J. (2016). Outbreak of Influenza A(H3N2) Variant Virus Infections Among Persons Attending Agricultural Fairs Housing Infected Swine—Michigan and Ohio, July–August 2016. MMWR Morb. Mortal. Wkly. Rep..

[10] Bowman A.S., Walia R.R., Nolting J.M., Vincent A.L., Killian M.L., Zentkovich M.M., Forshey T. (2017). Influenza A(H3N2) Virus in Swine at Agricultural Fairs and Transmission to Hu-mans, Michigan and Ohio, USA, 2016. Emerg. Infect. Dis..

[11] Rajão D.S., Gauger P.C., Anderson T.K., Lewis N.S., Abente E.J., Killian M.L., Perez D.R., Sutton T.C., Zhang J., Vincent A. (2015). Novel Reassortant Human-Like H3N2 and H3N1 Influenza A Viruses Detected in Pigs Are Virulent and Antigenically Distinct from Swine Viruses Endemic to the United States. J. Virol..

[12] Rajao D., Walia R., Campbell B., Gauger P.C., Janas-Martindale A., Killian M.L., Vincent A. (2016). Reassortment between Swine H3N2 and 2009 Pandemic H1N1 in the United States Resulted in Influenza A Viruses with Diverse Genetic Constellations with Variable Virulence in Pigs. J. Virol..

[13] Rajao D.S., Anderson T.K., Kitikoon P., Stratton J., Lewis N.S., Vincent A. (2018). Antigenic and genetic evolution of contemporary swine H1 influenza viruses in the United States. Virology.

[14] De Jong J.C., Van Nieuwstadt A.P., Kimman T.G., Loeffen W.L.A., Bestebroer T.M., Bijlsma K., Claas E.C.J. (1999). Antigenic drift in swine influenza H3 haemagglutinins with implica-tions for vaccination policy. Vaccine.

[15] Anderson T.K., Campbell B.A., Nelson M.I., Lewis N.S., Janas-Martindale A., Killian M.L., Vincent A.L. (2015). Characterization of co-circulating swine influenza A viruses in North Amer-ica and the identification of a novel H1 genetic clade with antigenic significance. Virus Res..

[16] Lorusso A., Vincent A., Harland M.L., Alt D., Bayles D.O., Swenson S.L., Gramer M.R., Russell C.A., Smith D.J., Lager K.M. (2010). Genetic and antigenic characterization of H1 influenza viruses from United States swine from 2008. J. Gen. Virol..

[17] Nelson M.I., Stucker K.M., Schobel S.A., Trovão N.S., Das S.R., Dugan V.G., Nelson S.W., Sreevatsan S., Killian M.L., Nolting J.M. (2016). Introduction, Evolution, and Dissemination of Influenza A Viruses in Exhibition Swine in the United States during 2009 to 2013. J. Virol..

[18] Nelson M.I., Wentworth E.D., Das S.R., Sreevatsan S., Killian M.L., Nolting J.M., Slemons R.D., Bowman A.S. (2015). Evolutionary Dynamics of Influenza A Viruses in US Exhibition Swine. J. Infect. Dis..

[19] Webby R.J., Rossow K., Erickson G., Sims Y., Webster R. (2004). Multiple lineages of antigenically and genetically diverse influenza A virus co-circulate in the United States swine population. Virus Res..

[20] Joseph U., Su Y.C.F., Vijaykrishna D., Smith G.J.D. (2016). The ecology and adaptive evolution of influenza A interspecies transmission. Influ. Other Respir. Viruses.

[21] Wong K.K., Greenbaum A., Moll M.E., Lando J., Moore E.L., Ganatra R., Biggerstaff M., Lam E., Smith E.E., Storms A.D. (2012). Outbreak of Influenza A (H3N2) Variant Virus Infection among Attendees of an Agricultural Fair, Pennsylvania, USA, 2011. Emerg. Infect. Dis..

[22] Jhung M.A., Epperson S., Biggerstaff M., Allen D., Balish A., Barnes N., Beaudoin A., Berman L., Bidol S., Blanton L. (2013). Outbreak of Variant Influenza A(H3N2) Virus in the United States. Clin. Infect. Dis..

[23] Bowman A.S., Nelson S.W., Page S.L., Nolting J.M., Killian M.L., Sreevatsan S., Slemons R.D. (2014). Swine-to-Human Transmission of Influenza A(H3N2) Virus at Agricultural Fairs, Ohio, USA, 2012. Emerg. Infect. Dis..

[24] World Health Organization (2016). WHO Tool for Influenza Pandemic Risk Assessment (TIPRA).

[25] Cox N., Trock S.C., Burke S.A. (2014). Pandemic Preparedness and the Influenza Risk Assessment Tool (IRAT). Curr. Top. Microbiol. Immunol..

[26] Nachbagauer R., Choi A., Hirsh A., Margine I., Iida S., Barrera A., Ferres M., Albrecht R.A., García-Sastre A., Bouvier N.M. (2017). Defining the antibody cross-reactome directed against the influenza virus surface glycoproteins. Nat. Immunol..

[27] Skowronski D.M., Janjua N.Z., De Serres G., Purych D., Gilca V., Scheifele D.W., Dionne M., Sabaiduc S., Gardy J.L., Li G. (2012). Cross-reactive and Vaccine-Induced Antibody to an Emerging Swine-Origin Variant of Influenza A Virus Subtype H3N2 (H3N2v). J. Infect. Dis..

[28] Epperson S., Jhung M., Richards S., Quinlisk P., Ball L., Moll M., Boulton R., Haddy L., Biggerstaff M., Brammer L. (2013). Human Infections With Influenza A(H3N2) Variant Virus in the United States, 2011–2012. Clin. Infect. Dis..

[29] Kitikoon P., Nelson M.I., Killian M.L., Anderson T.K., Koster L., Culhane M.R., Vincent A.L. (2013). Genotype patterns of contemporary reassorted H3N2 virus in US swine. J. Gen. Virol..

[30] Anderson T.K., Macken C.A., Lewis N.S., Scheuermann R.H., Van Reeth K., Brown I.H., Swenson S.L., Simon G., Saito T., Berhane Y. (2016). A Phylogeny-Based Global Nomenclature System and Automated Annotation Tool for H1 Hemagglutinin Genes from Swine Influenza A Viruses. mSphere.

[31] Chang J., Anderson T.K., Zeller M.A., Gauger P.C., Vincent A. (2019). octoFLU: Automated Classification for the Evolutionary Origin of Influenza A Virus Gene Sequences Detected in U.S. Swine. Microbiol. Resour. Announc..

[32] Zhang Y., Aevermann B.D., Anderson T.K., Burke D.F., Dauphin G., Gu Z., Scheuermann R.H. (2016). Influenza Research Database: An integrated bioinformatics resource for in-fluenza virus research. Nucleic Acids Res..

[33] Stamatakis A. (2014). RAxML version 8: A tool for phylogenetic analysis and post-analysis of large phylogenies. Bioinformatics.

[34] Katz J.M., Hancock K., Xu X. (2011). Serologic assays for influenza surveillance, diagnosis and vaccine evaluation. Expert Rev. Anti-Infect. Ther..

[35] Yen H.-L., Webster R.G. (2009). Pandemic Influenza as a Current Threat. Curr. Top. Microbiol. Immunol..

[36] Miller E., Hoschler K., Hardelid P., Stanford E., Andrews N., Zambon M. (2010). Incidence of 2009 pandemic influenza A H1N1 infec-tion in England: A cross-sectional serological study. Lancet.

[37] (2018). Annual Estimates of the Resident Population by Single Year of Age and Sex for the United States: 1 April 2010 to 1 July 2018.

[38] Verma N., Dimitrova M., Carter N.M., Crevar C.J., Ross T.M., Golding H., Khurana S. (2012). Influenza Virus H1N1pdm09 Infections in the Young and Old: Evidence of Greater Antibody Diversity and Affinity for the Hemagglutinin Globular Head Domain (HA1 Domain) in the Elderly than in Young Adults and Children. J. Virol..

[39] Lewis N. (2016). (Nicola); A Russell, C.; Langat, P.; Anderson, T.K.; Berger, K.; Bielejec, F.; Burke, D.; Dudas, G.; Fonville, J.M.; Fouchier, R.A.; et al. Author response: The global antigenic diversity of swine influenza A viruses. Author Response.

[40] Webster R.G. (1966). Original antigenic sin in ferrets: The response to sequential infections with influenza viruses. J. Immunol..

