# Supplementary material for: Gaps in Serologic Immunity against Contemporary Swine-Origin Influenza A Viruses among Healthy Individuals in the United States
Source: Viruses. 2021 Jan 18;13(1):127. doi: 10.3390/v13010127 (PMC7830885; doi:10.3390/v13010127)
Supplement: Supplementary file 1 [file viruses-13-00127-s001.pdf]

# Supplementary Materials for

## Gaps in serologic immunity against contemporary swine-origin influenza A viruses among healthy individuals in the United States

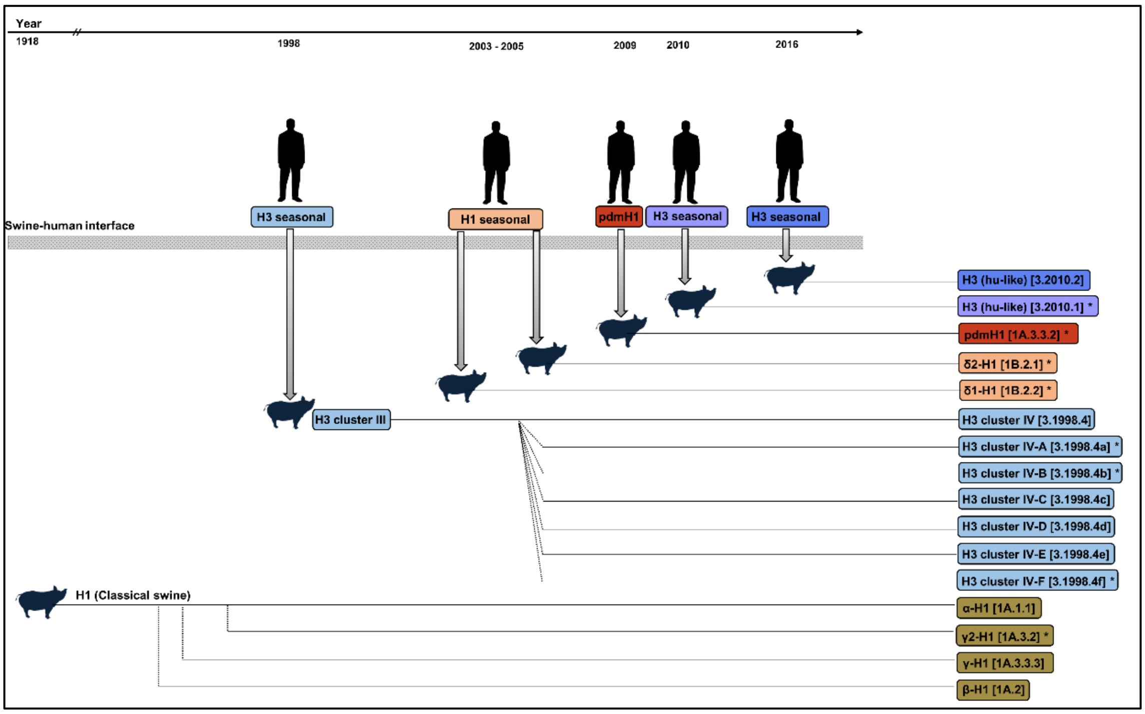


**Supplementary Figure 1.** Contemporary United States Swine Influenza A Virus H1 and H3 Hemagglutinin Segments. Distinct HA subclades represented in contemporary influenza A virus (IAV) found in the US swine herd are shown with both colloquial and phylogeny-based global nomenclature (brackets) at right. Colors highlight introduction events leading to establishment of stable IAV lineages in swine. Gray block arrows connecting human and pig silhouettes represent establishment of HA segments in swine following reverse-zoonotic transmission. Broken (dashed, dotted) lines indicate evolution events resulting in novel phylogenetic subclades.

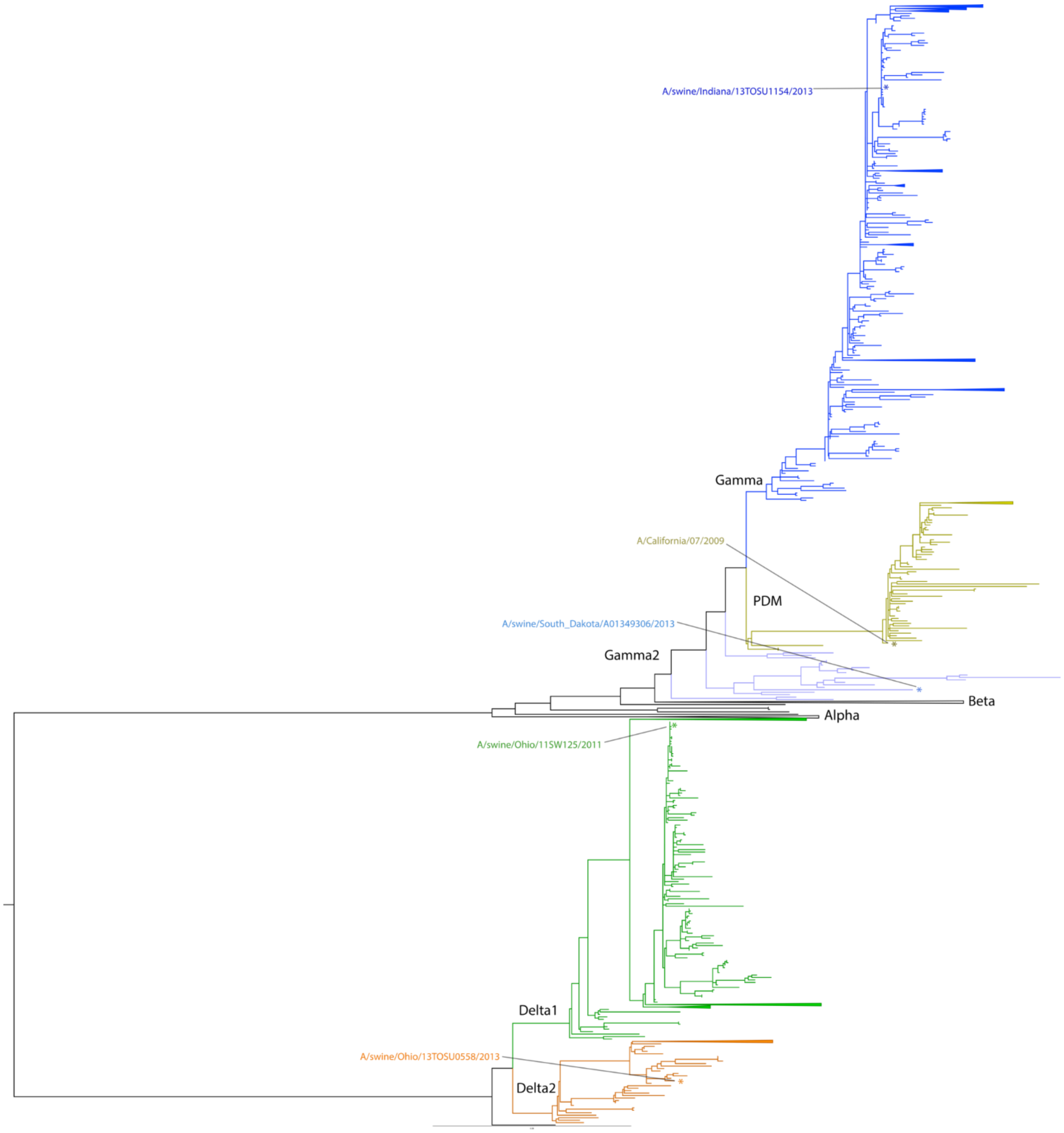


**Supplementary Figure 2.** Phylogenetic relationships of hemagglutinin (HA) sequences for H1-subtype IAV study strains. H1 sublineages are indicated and distinguished by distinct colors. Tip labels are provided, and study strain names are colored to match the corresponding sublineage. Scale bar (bottom) indicates nucleotide substitutions per site.

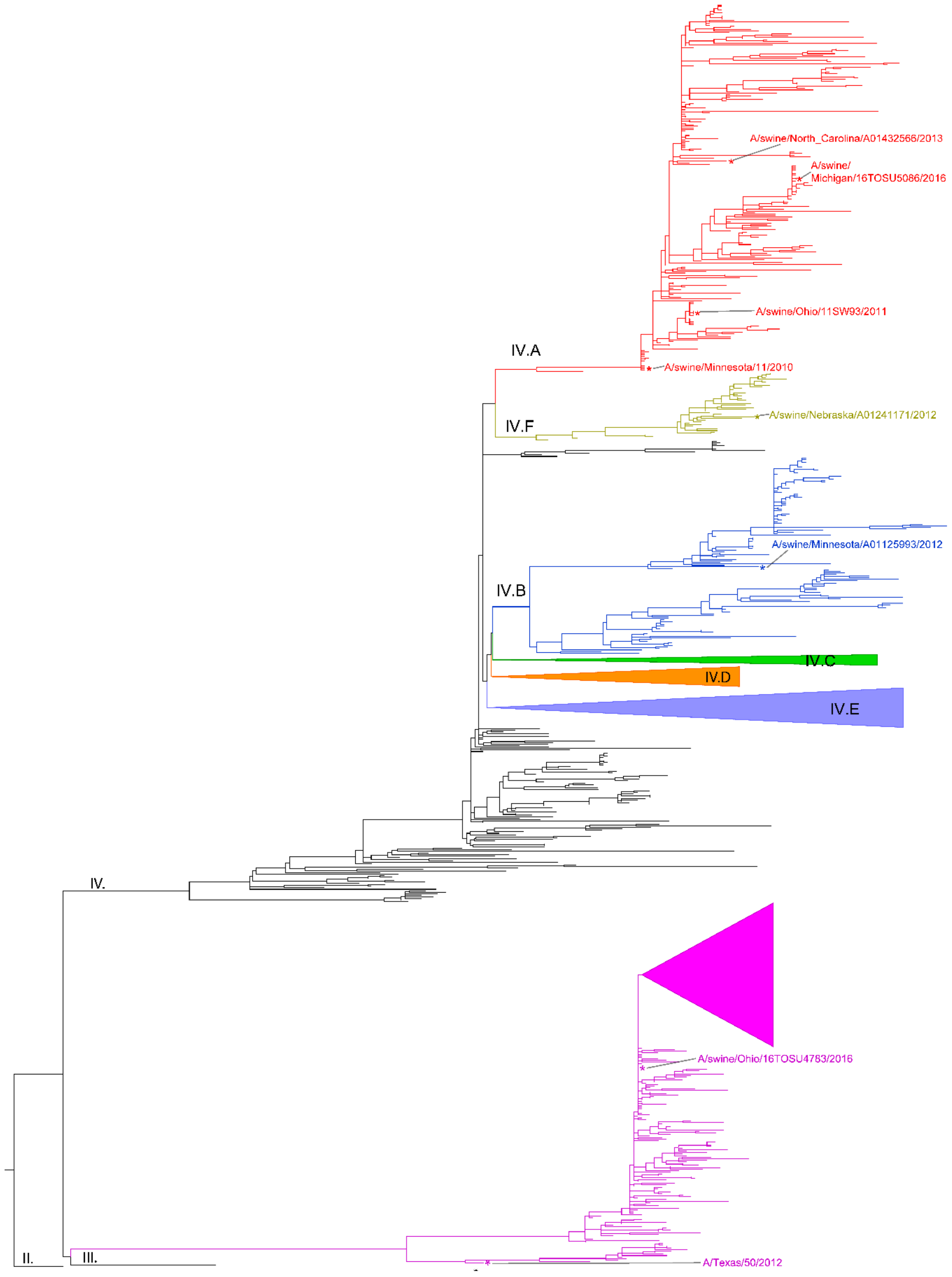


**Supplementary Figure 3**. Phylogenetic relationships of hemagglutinin (HA) sequences for H3-subtype IAV study strains. H3 sublineages are indicated and distinguished by distinct colors. Tip labels are provided, and study strain names are colored to match the corresponding sublineage. Scale bar (bottom) indicates nucleotide substitutions per site.

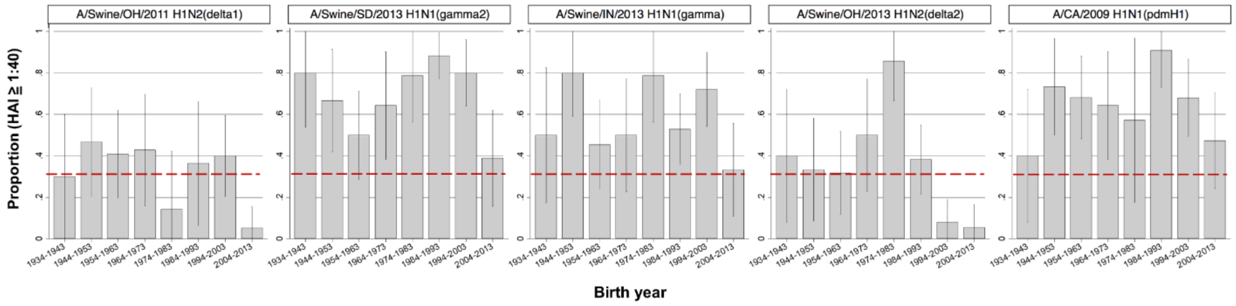


**Supplementary Figure 4.** Group seroprotection rate (8 decades of birth) for H1 subtype IAVs. Proportion of individuals with HAI antibody titer ≥ 1:40 (seroprotection rate; SPR) is shown for sequential decades of birth (n=8). Dashed red line at y= 0.3 corresponds to SPR of 30%. Spikes and caps indicate 95% confidence interval of the proportion.

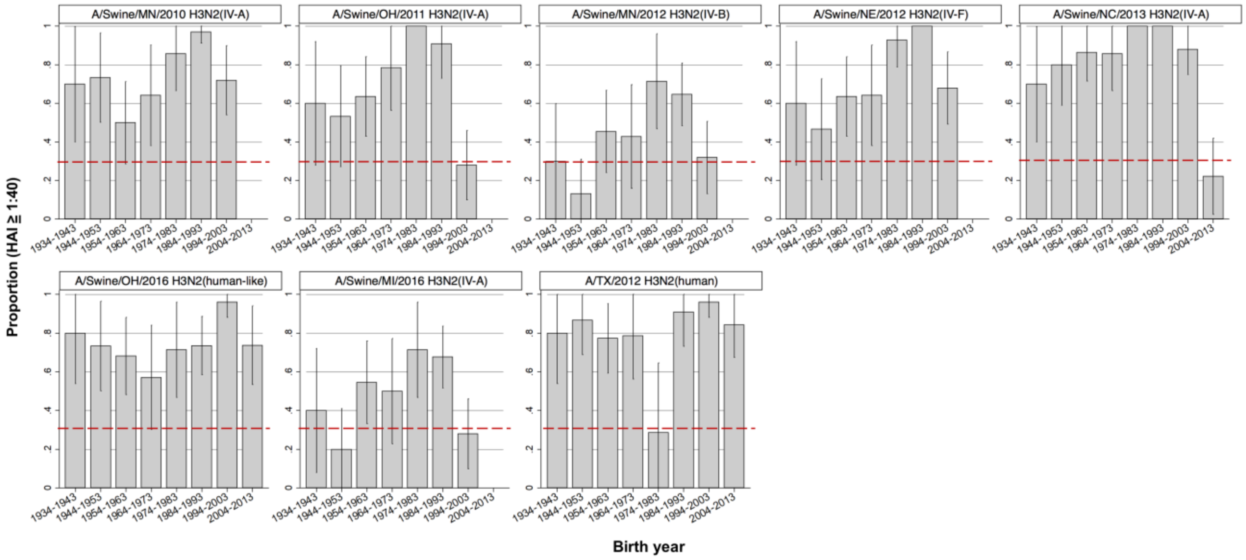


**Supplementary Figure 5.** Group seroprotection rate (8 decades of birth) for H3 subtype IAVs. Proportion of individuals with HAI antibody titer ≥ 1:40 (seroprotection rate; SPR) is shown for sequential decades of birth (n=8). Dashed red line at y= 0.3 corresponds to SPR of 30%. Spikes and caps indicate 95% confidence interval of the proportion.

**Supplementary Table 1.** Study virus nomenclature and GenBank accession numbers (hemagglutinin segment).

| Virus name, short | Virus name, full | Subtype | HA subclade nomenclature Colloquial Global [a] | Genbank accession |
|---|---|---|---|---|
| A/Swine/IN/2013 | A/Swine/IN/13TOSU1154/2013(H1N1) | H1N1 | gamma 1A.3.3.3 | KJ640612 |
| A/Swine/SD/2013 | A/Swine/SD/A01349306/2013(H1N1) | H1N1 | gamma2 1A.3.2 | KC844200 |
| A/CA/2009 | A/CA/07/2009(H1N1) | H1N1 | pdmH1 1A.3.3.2 | GQ280797 |
| A/Swine/OH/2011 | A/Swine/OH/11SW125/2011(H1N2) | H1N2 | delta1 1B.2.2 | CY131349 |
| A/Swine/OH/2013 | A/Swine/OH/13TOSU0558/2013(H1N2) | H1N2 | delta2 1B.2.1 | KJ640371 |
| A/TX/2012 | A/TX/50/2012(H3N2) | H3N2 | human- | KC892952 |
| A/Swine/OH/2016 | A/Swine/OH/16TOSU4783/2016(H3N2) | H3N2 | 2010 human-like 3.2010.1 | KX981534 |
| A/Swine/MI/2016 | A/Swine/MI/16TOSU5086/2016(H3N2) | H3N2 | C_IV-A 3.1990.4.a | MN708371 |
| A/Swine/MN/2010 | A/Swine/MN/11/2010(H3N2) | H3N2 | C_IV-A 3.1990.4.a | KJ942624 |
| A/Swine/NC/2013 | A/Swine/NC/A01432566/2013(H3N2) | H3N2 | C_IV-A 3.1990.4.a | KC841842 |
| A/Swine/OH/2011 | A/Swine/OH/11SW93/2011(H3N2) | H3N2 | C_IV-A 3.1990.4.a | CY13127 |
| A/Swine/MN/2012 | A/Swine/MN/A01125993/2012(H3N2) | H3N2 | C_IV-B 3.1990.4.b | JX422257 |
| A/Swine/NE/2012 | A/Swine/NE/A01241171/2012(H3N2) | H3N2 | C_IV-F 3.1990.4.f | JX422575 |

[a] HA subclade global nomenclature is based on recent suggested guidelines for naming H1-subtype and H3-subtype IAVs in swine [21,22].

**Supplementary Table 2.** Number of serum samples analyzed by HAI [a] assay within individual age groups [b] and overall.

| Virus | Subclade | Juvenile [c] | Adult [d] | Senior [e] | Overall |
|---|---|---|---|---|---|
| A/Swine/OH/2011 (H1N2) | delta1 | 44 | 24 | 55 | 123 |
| A/Swine/OH/2013 (H1N2) | delta2 | 44 | 54 | 55 | 152 |
| A/Swine/IN/2013 (H1N1) | gamma | 43 | 54 | 55 | 152 |
| A/Swine/SD/2013 (H1N1) | gamma2 | 43 | 54 | 55 | 152 |
| A/CA/2009 (H1N1) | pdmH1 | 44 | 24 | 55 | 123 |
| A/Swine/MN/2010 (H3N2) | C_IV-A | 44 | 54 | 55 | 153 |
| A/Swine/OH/2011 (H3N2) | C_IV-A | 44 | 24 | 55 | 123 |
| A/Swine/NC/2013 (H3N2) | C_IV-A | 43 | 54 | 55 | 152 |
| A/Swine/MI/2016 (H3N2) | C_IV-A | 44 | 54 | 55 | 153 |
| A/Swine/MN/2012 (H3N2) | C_IV-B | 43 | 54 | 55 | 152 |
| A/Swine/NE/2012 (H3N2) | C_IV-F | 43 | 54 | 55 | 152 |
| A/Swine/OH/2016 (H3N2) | human-like | 44 | 54 | 55 | 153 |
| A/TX/2012 (H3N2) | human | 44 | 24 | 55 | 123 |

[a] Hemagglutination inhibition; [b] Age groups constructed based on those utilized in the Tool for Influenza Pandemic Risk Assessment (TIPRA); [c] Individuals < 18 years old; [d] Individuals 18-49 years old; [e] Individuals ≥ 50 years old.
